# Peptide 1018 inhibits swarming and influences Anr-regulated gene expression downstream of the stringent stress response in *Pseudomonas aeruginosa*

**DOI:** 10.1371/journal.pone.0250977

**Published:** 2021-04-30

**Authors:** Lauren V. Wilkinson, Morgan A. Alford, Shannon R. Coleman, Bing C. Wu, Amy H. Y. Lee, Travis M. Blimkie, Manjeet Bains, Reza Falsafi, Daniel Pletzer, Robert E. W. Hancock

**Affiliations:** 1 Centre for Microbial Diseases and Immunity Research, Department of Microbiology and Immunology, University of British Columbia, Vancouver, Canada; 2 Department of Molecular Biology and Biochemistry, Simon Fraser University, Burnaby, Canada; 3 Department of Microbiology and Immunology, University of Otago, Dunedin, New Zealand; Laurentian University, CANADA

## Abstract

*Pseudomonas aeruginosa* is a ubiquitous opportunistic pathogen that causes considerable human morbidity and mortality, particularly in nosocomial infections and individuals with cystic fibrosis. *P*. *aeruginosa* can adapt to surface growth by undergoing swarming motility, a rapid multicellular movement that occurs on viscous soft surfaces with amino acids as a nitrogen source. Here we tested the small synthetic host defense peptide, innate defense regulator 1018, and found that it inhibited swarming motility at concentrations as low as 0.75 μg/ml, well below the MIC for strain PA14 planktonic cells (64 μg/ml). A screen of the PA14 transposon insertion mutant library revealed 29 mutants that were more tolerant to peptide 1018 during swarming, five of which demonstrated significantly greater swarming than the WT in the presence of peptide. Transcriptional analysis (RNA-Seq) of cells that were inoculated on swarming plates containing 1.0 μg/ml peptide revealed differential expression of 1,190 genes compared to cells swarming on plates without peptide. Furthermore, 1018 treatment distinctly altered the gene expression profile of cells when compared to that untreated cells in the centre of the swarm colonies. Peptide-treated cells exhibited changes in the expression of genes implicated in the stringent stress response including those regulated by *anr*, which is involved in anaerobic adaptation, indicative of a mechanism by which 1018 might inhibit swarming motility. Overall, this study illustrates potential mechanisms by which peptide 1018 inhibits swarming surface motility, an important bacterial adaptation associated with antibiotic resistance, virulence, and dissemination of *P*. *aeruginosa*.

## Introduction

The accelerated emergence of antibiotic resistance mechanisms in bacterial pathogens threatens our ability to treat major, and even minor, infections. Intrinsic and adaptive properties of some pathogenic species of bacteria contribute to their resistance in the clinic. For example, the opportunistic pathogen *Pseudomonas aeruginosa* has an outer membrane permeability 12–100 times less than that of *Escherichia coli* and an impressive suite of multidrug efflux pumps [1–3]. Adaptability is associated with transient growth states, including biofilm formation and swarming motility, in which many genes change expression, including those leading to multidrug resistance [3–6]. *P*. *aeruginosa* adaptability is reflected by its pathogenicity in diverse areas of the human body; it can cause acute and/or chronic infections in the lungs, burn wounds, urinary tract and bloodstream [7–9]. Swarming motility manifests as a form of rapid surface translocation in which groups of cells move coordinately using both flagella and pili to propel themselves, assisted by production of the surface-wetting agent rhamnolipids [10]. Additionally, swarming motility is involved in bacterial disemmination in a murine abscess model [11] and *P*. *aeruginosa* exhibits increased resistance to most conventional antibiotics during swarming motility [3, 12]. The mechanism underlying swarm-associated multi-drug resistance is not fully understood but is independent of efflux pump activity and synthesis of periplasmic glucans, which contribute to resistance in planktonic and biofilm growth states respectively [13].

Biofilm and swarming growth states rely on the stringent stress response signaling pathway that is triggered by the alarmone (p)ppGpp, levels of which increase under the nutrient-limiting or anoxic conditions that are characteristic of chronic infection sites [14, 15]. In turn, bacteria downregulate the expression of genes involved in central metabolism and divert cells toward a growth arrest phenotype [14, 15]. The stringent stress response, however, is also functional under optimal growth conditions [14]. In fact, swarming motility and the production of rhamnolipids, which can occur in oxygen- and nutrient-replete media, are dependent on the stringent-stress response [10, 14, 16].

Innate defense regulator 1018 is a host-defense peptide derived from bovine bactenecin [17]. Host-defense peptides with multifaceted mechanisms of action have received increasing attention for their potential as adjuvants for conventional antibiotic therapies [18, 19]. Numerous studies have investigated the anti-biofilm properties of a subset of these peptides and the clinical utility of peptide derivatives (reviewed in ref. 18). Peptide 1018 has a broad range of beneficial immunomodulatory effects, including suppression of lipopolysaccharide-induced macrophage-mediated inflammatory responses, and antimicrobial effects in various infection models [11, 19, 20], as well as broad-spectrum activity against biofilms [21] through a mechanism dependent on the stringent stress response. Interestingly, it has more potent antibiofilm than antibacterial activity, with a minimal biofilm inhibitory concentration (MBIC) of 2–10 μg/ml, cf. an MIC of 64 μg/ml for strain PA14 [21]. We tested here its activity against another adaptive growth state, swarming motility.

To identify how peptide 1018 inhibited swarming at a remarkably low concentration (0.75 μg/ml), we compared the transcriptome of cells inoculated onto swarming plates containing 1018 to that of cells swarming in the absence of peptide. We identified modulation of metabolic adaptations implicated in the stringent stress response. This modulation was at least partially mediated by the metabolic regulator Anr, since 114 of 253 genes in the Anr regulon were differentially expressed following peptide treatment. Furthermore, the transcriptional profile of cells inoculated on swarming plates with peptide was different from that of quiescent cells in the centre of swarming colonies inoculated on plates without peptide. Swarming in the presence of peptide was restored by the addition of serine hydroxamate (SHX), which promotes (p)ppGpp synthesis, providing evidence that 1018 works at least in part through the stringent stress response.

## Materials and methods

### Bacterial strains and plasmids

The PA14NR library, consisting of 5,850 transposon insertion mutants corresponding to 4,596 predicted PA14 genes [22], was used to screen for mutants that swarmed in the presence of peptide 1018. Phenotypes are described relative to that of the PA14 wild type (WT). All bacterial strains used in this study are listed in S1 Table.

### Growth conditions

Bacterial strains were grown overnight in Luria-Bertani (LB) broth at 37°C with aeration. Unless specified, overnight cultures were sub-cultured into either LB broth or basal medium 2 (BM2) [62 mM potassium phosphate buffer (pH 7), 7 mM (NH_4_)_2_SO_4_, 2 mM MgSO_4_, 10 μM FeSO_4_, 0.4% (wt/vol) glucose] at a normalized optical density at 600 nm (OD_600_) of 0.1 and grown to mid-log phase (OD_600_ of 0.4–0.6) at 37°C with aeration. 15 μg/ml gentamicin was included when required for transposon selection and maintenance.

### Peptide

Peptide 1018 was dissolved in distilled water, aliquoted and stored at -20°C until use. All experiments were performed using batch CL-03-00140 of peptide 1018 (>95% purity), synthesized by CPC Scientific using solid-phase 9-fluorenylmethoxy carbonyl (Fmoc) chemistry and purified using reverse-phase high-performance liquid chromatography (HPLC).

### Broad library swarming screen

A high throughput swarming screen was modified from Overhage *et al*. [23] to examine the inhibition of swarming motility by peptide 1018 in the PA14NR transposon insertion mutant library. Bacteria were grown in LB overnight in 96-well plates. A custom-made 96-pin stamp was used to transfer approximately 1 μl of overnight culture onto agar plates containing BM2 glucose swarming medium [62 mM potassium phosphate buffer (pH 7), 2 mM MgSO_4_, 10 μM FeSO_4_, 0.4% (wt/vol) glucose, 0.1% (wt/vol) Casamino acids (CAA), and 0.5% (wt/vol) Difco agar] [23]. Peptide 1018 was incorporated directly into the agar at 1 μg/ml. All swarming plates were incubated at 37°C for 18–20 h. Colonies were visually assessed for their ability to swarm in the presence of the peptide. Two to three replicates were conducted for each plate of mutants in the PA14NR library.

### Standard swarming assay

The standard swarming assay for *P*. *aeruginosa* involved inoculating 1 μl of mid-log phase sub-culture into the centre of a polystyrene plate containing BM2 glucose swarming agar supplemented with 0.1% CAA. This assay was used to verify the results of the broad library screen and subsequent experiments. When necessary, peptide 1018 (0–2 μg/ml) or serine hydroxamate (SHX) (0–1000 μM) were incorporated directly into the agar at concentrations indicated in the Figure legends. Plates were incubated at 37°C for 18–20 h. At the endpoint, an image of each plate was captured using a BioRad ChemiDoc, and the surface area of each swarming colony was quantified using ImageJ software (v1.8.0; Redwood, CA, USA). Each swarming assay was carried out three to five times.

### Microscopy

Transmission electron microscopy (TEM) was used to investigate differences in cellular morphology between untreated cells from the leading edge of the swarm front and swarm centre, when compared to peptide-treated cells. The TEM protocol was modified from Köhler *et al*. [15]. Briefly, cells were picked with a sterile pipette tip and gently resuspended in 10 μl water. Formvar and carbon-coated copper TEM grids (200-mesh) were placed on top of the suspension for 30 s to allow cell adherence. Excess liquid was removed using filter paper. The grids were stained with 5 μl of a 2% aqueous uranyl acetate solution for 30 s and then washed for 5 s in 10 μl water. Excess liquid was removed from the grids with filter paper, and they were allowed to air dry. Images from multiple grid sections were taken with a Hitachi H7600 TEM at the UBC Bioimaging facility.

### RNA isolation

One μl of PA14 WT mid-log phase sub-culture was inoculated onto the centre of each BM2 swarming agar plate, with or without 1 μg/ml peptide 1018. Plates were incubated for 20 h at 37°C, after which cells were harvested for RNA isolation. Cells were collected in RNAprotect Bacteria Reagent (Qiagen) with sterile swabs from the leading 2–3 mm of the swarming edge, and centres of swarming colonies grown on standard BM2 swarming plates. The entire colony was collected from plates treated with peptide 1018. Cells from three independent biological replicates in each condition were harvested. Total RNA extracted from these cells by resuspending in 100 μl of 3 mg/ml lysozyme in Tris-EDTA (TE) buffer (pH 8.0; Thermo Fisher), followed by extraction using the RNeasy Mini kit (Qiagen) according to the manufacturer’s protocol. Purified RNA underwent deoxyribonuclease (DNase) treatment with the TURBO DNA-free kit (Ambion) to remove chromosomal DNA. RNA purity was assessed using the Agilent 2100 Bioanalyzer.

### RNA-Seq and identification of DE genes

To enrich for mRNA, we performed rRNA depletion on the purified total RNA with the RiboZero Bacteria kit (Illumina), followed by the Kapa stranded Total RNA kit (Kapa Biosystems) and the indexing kit (Bio Scientific, USA) to construct multiplexed cDNA libraries for sequencing. The libraries were sequenced on an Illumina HiSeq 2500 platform in one lane of a high-output flowcell to generate 100-bp single-end reads at the UBC Sequencing and Bioinformatics Consortium (SBC). Subsequently, FastQC v0.11.5 and MulitQC v0.8 were used to obtain the quality score for FASTQ files [24]. STAR aligner was used to align reads to the UCBPP-PA14 genome (GCF_000014625.1) [25, 26]. Read count tables were then generated with HTseq-count v0.6.1p1 [27]. DESeq2 v1.18 was used to estimate the fold-change (FC) in gene expression between untreated cells from the edge or centre and peptide-treated cells under swarming conditions [28] with thresholds of adjusted *P* ≤ 0.05 and absolute FC ≥ 1.5. The list of differentially expressed (DE) genes is available in S2 Table. RNA-Seq data were deposited in NCBI GEO under the accession number GSE151264.

### Functional enrichment of DE genes

Enrichment of gene ontology (GO) terms was performed using the R package GofuncR [29], testing the DE genes against a custom set of GO annotations downloaded from the *Pseudomonas* genome database [30]. Results were filtered using a significance-threshold of *P* ≤ 0.05 and a multi-test corrected family-wise error rate (FWER) ≤ 0.1. The full list of functionally enriched GO terms is available in S3 Table. DE genes belonging to the *anr* regulon [31] in the 1018 vs. edge comparison were tested for enrichment using Fisher’s Exact test. All analyses described were performed in R (v4.0.3; Boston, MA, United States).

### Statistical analysis

Unless otherwise stated, statistics were performed using GraphPad Prism 8.0 (La Jolla, CA, United States). *P*-values were calculated using one-way ANOVA followed by Dunnett’s correction, Kruskal–Wallis nonparametric test followed by Dunn’s post-hoc analysis, or Student’s two-tailed *t*-test as indicated in the Figure legends. Data were considered statistically significant when *P* ≤ 0.05.

## Results

### 1018 inhibited swarming motility in *P*. *aeruginosa*

**Fig 1:**
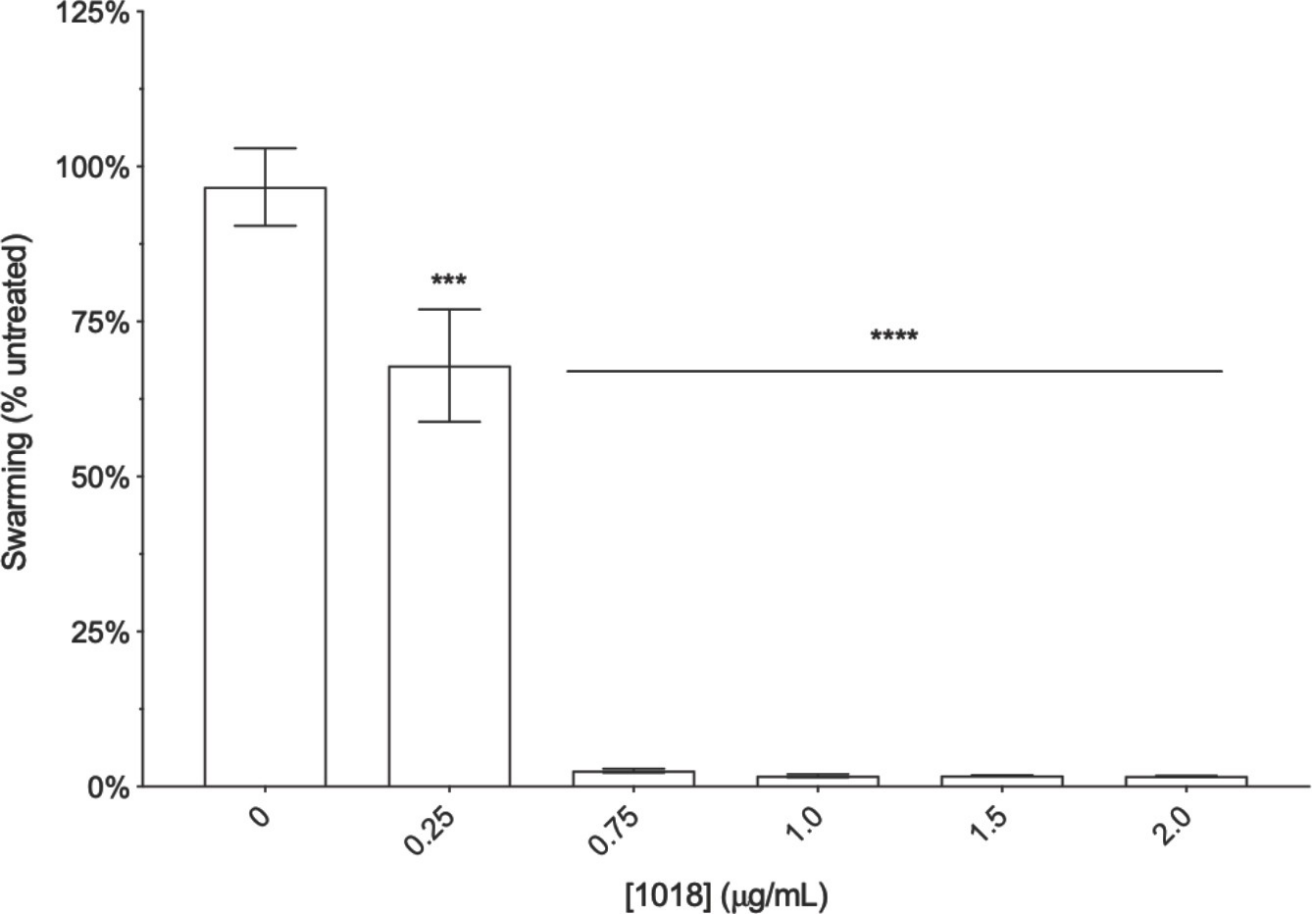
Peptide 1018 inhibited swarming motility in PA14 at low concentrations. BM2 swarm plates containing 1018 or water only were inoculated with 1 μl of planktonic cells suspended at an OD_600_ of 0.4–0.6 in BM2 and then incubated for 20 h at 37°C. Mean and standard error of the mean are shown. One-way ANOVA followed by Dunn’s correction was used to determine statistical significance. *** *P* < 0.001, **** *P* < 0.0001. Each result was obtained from at least three independent biological replicates.

Titration of 1018 (0–2 μg/ml) into swarming agar revealed that this peptide inhibited swarming motility of *P*. *aeruginosa* strain PA14 at low concentrations (Fig 1). Swarming motility was significantly reduced by 28.8% when cells were inoculated on plates containing as little as 0.25 μg/ml 1018 when compared to cells inoculated on plates without peptide (0 μg/ml). In subsequent experiments, 0.75 or 1.0 μg/ml 1018 was used since it reduced swarming by 94.1 and 95.0% respectively.

**Fig 2:**
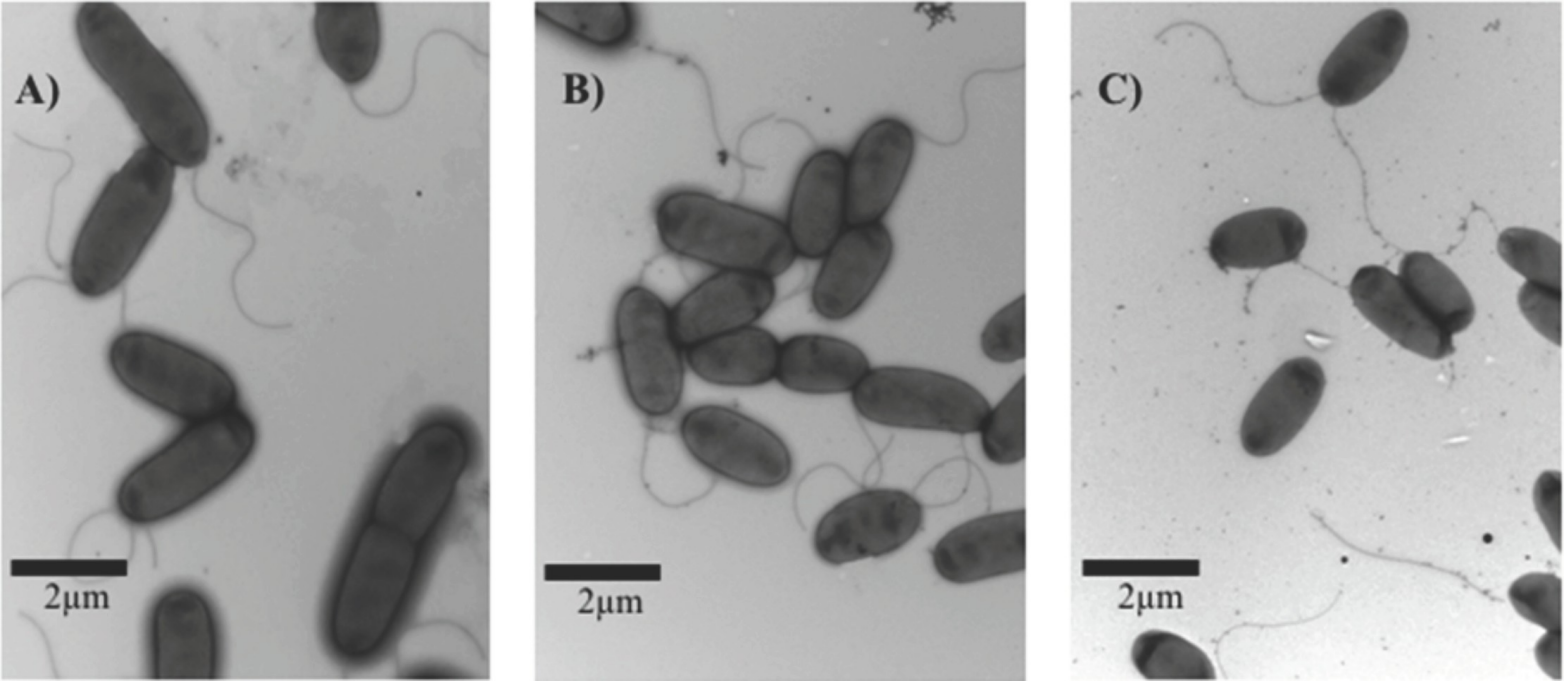
Representative images of cells captured using Transmission Electron Microscopy (TEM). Briefly, BM2 swarm plates (±0.75 μg/ml 1018) were inoculated with 1 μl of planktonic cells suspended at an OD_600_ of 0.4–0.6 in BM2 and then incubated for 20 h at 37°C. Cells were transferred to a mesh grid for TEM. (A) Actively swarming WT cells from the edge of a swarming colony. (B) Non-swarming cells taken from the centre of a swarming colony. (C) Cells from a swarming plate treated with 0.75 μg/ml of 1018.

Strain PA14 was harvested from peptide-containing swarm plates and examined by TEM (Fig 2). TEM revealed that bacteria from swarming plates with peptide 1018 were flagellated and resembled cells taken from the centre of untreated swarming colonies (Fig 2). More specifically, cells from the leading edge of the untreated swarm front were elongated and septated, indicative of cellular division, whereas cells from the centre of the untreated swarm colony and peptide-treated cells were neither elongated or septated.

### Twenty-nine transposon mutants swarmed in the presence of peptide 1018

**Fig 3:**
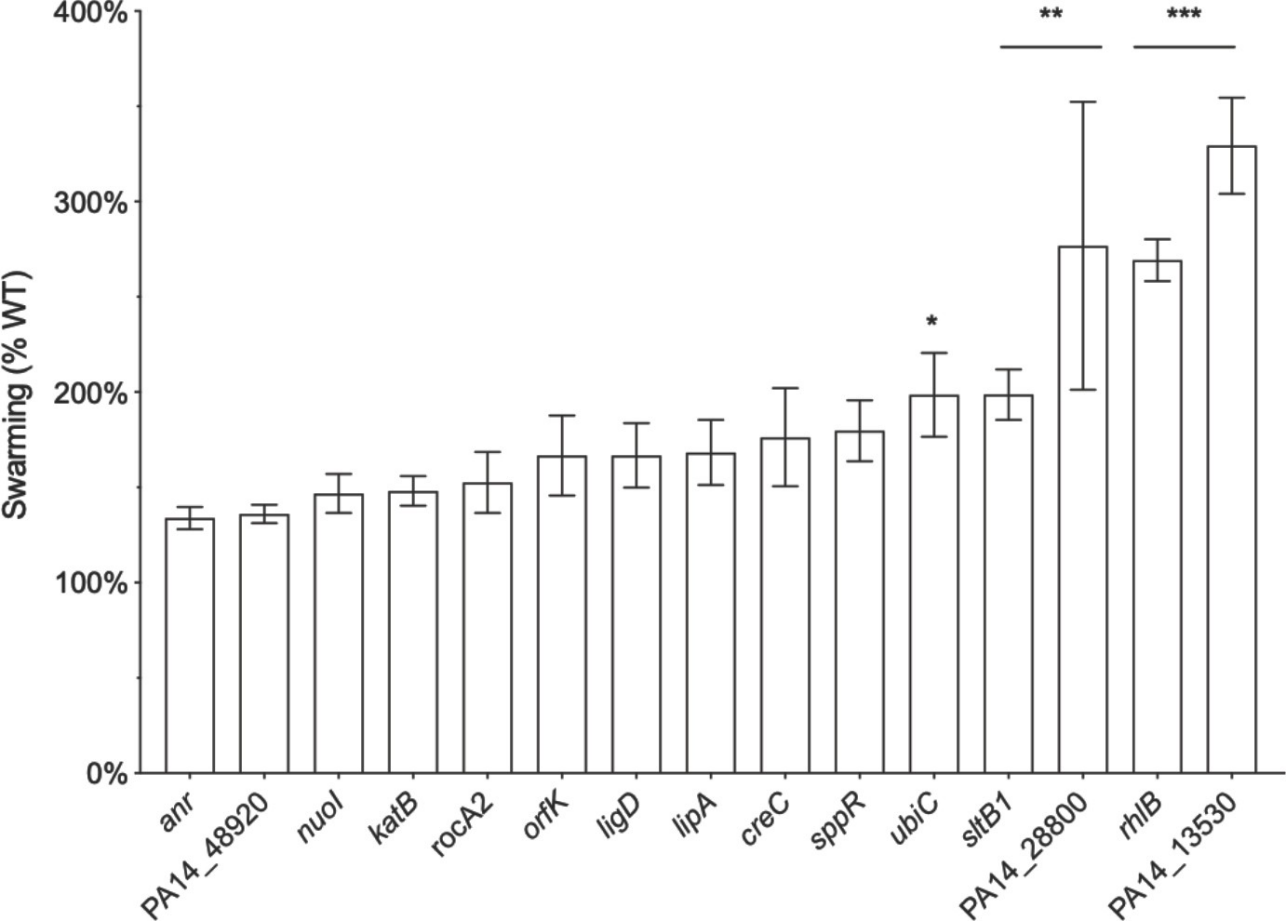
Selected PA14 transposon mutants that swarmed in the presence of 0.75 μg/ml peptide 1018. Briefly, BM2 swarm plates (with 0.75 μg/ml 1018) were inoculated with 1 μl of planktonic cells suspended at an OD_600_ of 0.4–0.6 in BM2 and then incubated for 20 h at 37°C. At least three biological replicates for each transposon mutant were examined. Mean and standard error of the mean are shown. * *P* < 0.05, ** *P* < 0.01, *** *P* < 0.001 according to Kruskal–Wallis test followed by Dunnett’s post hoc analysis.

The entire PA14 transposon insertion mutant library [22] was screened for swarming in the presence of an inhibitory concentration of 1018 (1 μg/ml). This screen revealed 29 transposon mutants that upon retesting were tolerant to peptide 1018 under swarming conditions. Five of these exhibited significantly increased (2.0- to 3.3-fold) swarming when compared to WT (Fig 3). Nonetheless, these peptide tolerant mutants swarmed 75% less than that of the untreated WT. In particular, mutations in genes implicated in the stringent stress response (*creC*, *lipA*) [32, 33], metabolism (*anr*) [31, 34] and rhamnolipid biosynthesis (*rhlB*) also conferred moderate peptide tolerance under swarming conditions that could be complemented with the respective gene in the transposon mutants (S1 Fig). Furthermore, transposon interruption of genes directly involved in coping with stress, including the hydrogen peroxide-inducible *katB*, *creC*, *lipA*, and *sppR*, conferred tolerance to peptide under swarming conditions. Other peptide-tolerant mutants had transposon insertions in genes that belonged to diverse functional categories including regulatory genes (*orfK* and PA3045), and genes involved in iron acquisition and virulence factor production (Fig 3).

### The transcriptome of peptide-treated cells was distinct from that of swarming cells

**Fig 4:**
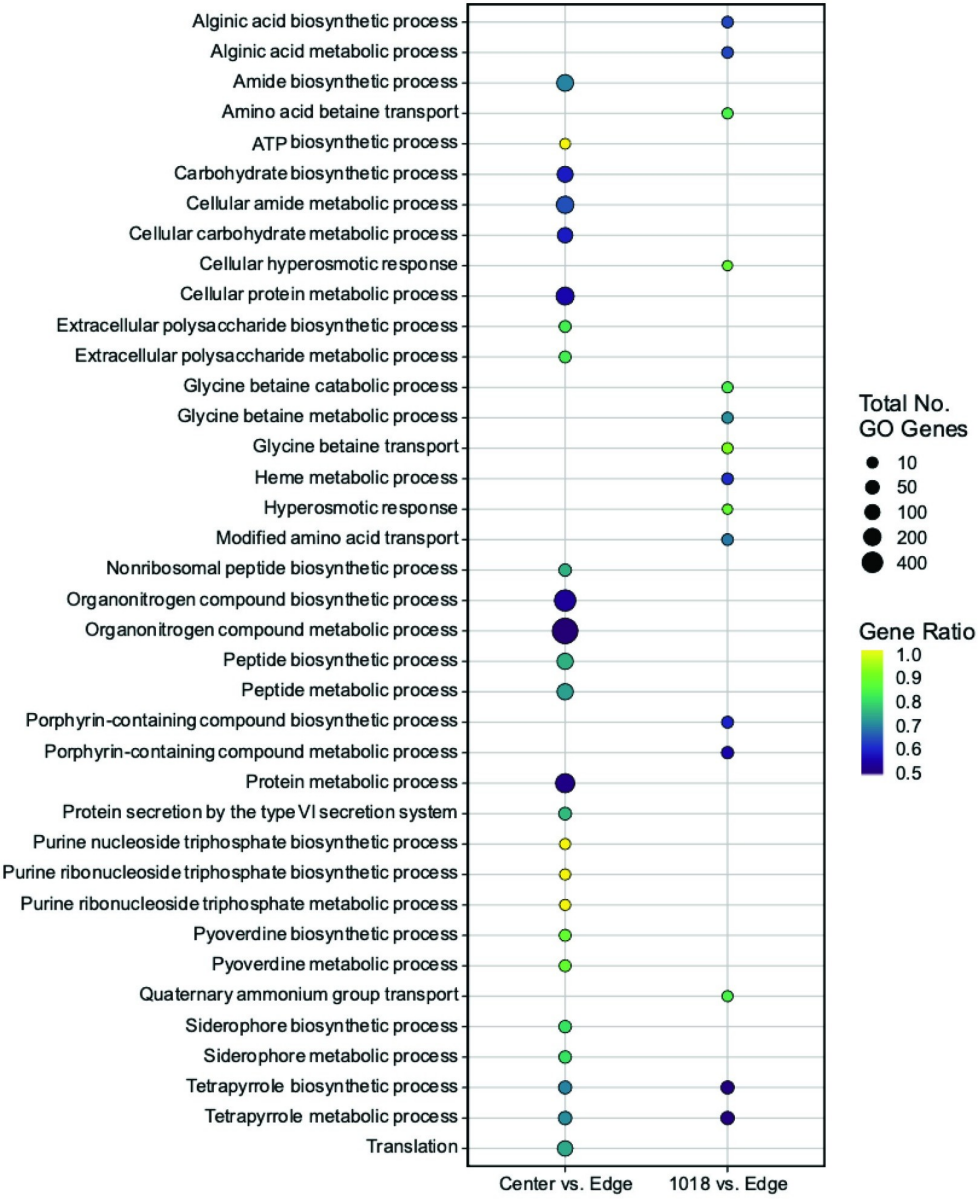
Functional classification of Differentially Expressed (DE) genes using Gene Ontology (GO) term enrichment. GO term enrichment was performed using GofuncR on the list of DE genes in cells treated with 1018 and cells at the centre of swarming colonies relative to cells at the edge of swarming colonies. GO terms were considered significant with a *P* ≤ 0.05 and a multi-test corrected family-wise error rate (FWER) ≤ 0.1. Cells treated with 1018, but not cells at the centre of swarming colonies, cf. swarming edge cells, exhibited functional enrichment in transport of small molecules, heme metabolism and amino acid biosynthesis/metabolism.

To characterize the cellular or molecular mechanisms by which 1018 influenced swarming motility, RNA-Seq was performed. The transcriptome of cells inoculated on swarm plates containing peptide (1.0 μg/ml) was compared to that of cells at the swarm front of colonies inoculated on plates without peptide (1018 vs. edge). The transcriptome of cells taken from the centre of colonies inoculated on peptide-free swarm plates was also compared to that of cells at the swarm front of the same plates (centre vs. edge). Peptide treatment resulted in the differential expression of 1,190 genes (S2 Table). DE genes in both treatment groups were functionally mapped according to gene ontology (Fig 4). Relative to swarming cells from the swarm leading edge, there was enrichment for DE genes encoding proteins involved in the transport of small molecules, heme metabolism and biosynthesis and metabolism of specific amino acids. Non-swarming cells from the swarm centre differentially expressed 2,369 genes when compared to cells from the swarm edge (S2 Table). In both peptide-treated and non-swarming cells, ~68% of all DE genes overlapped (S2 Table). However, a statistical test showed significant dysregulation of the *anr* regulon (*P* = 3.86E-18). As well, regulators of swarming and virulence were expressed differently in the two groups (Table 1) [31, 35]. Of note, *rhlA* and *rhlB* were 3-fold less upregulated in peptide-treated cells than untreated swarming cells when compared to the control.

**Table 1:** Selected categories of DE genes, mapped to PAO1 orthologs, as determined by RNA-Seq in peptide-treated and swarming colony centre cells under swarming conditions.

PAO1 locus (*name*)	Product	FC in gene expression	
		1018 treated vs. edge	Centre vs. edge
**Subset of *anr* regulon genes [31] (of the 141 DE genes in S4 Table)**			
PA0141	hypothetical protein	3.5	-2.6
PA0200	hypothetical protein	3.3	-2.2
PA0201	hypothetical protein	NC	-12
PA0408 (*pilG*)	twitching motility protein	NC	-1.7
PA0459 (*clpC*)	ClpA/B protease ATP binding subunit	5.3	1.9
PA0482 (*glcB*)	malate synthase G	1.6	NC
PA0519 (*nirS*)^**D**^	nitrite reductase	NC	-18
PA0524 (*norB*)^**D**^	nitric-oxide reductase subunit B	NC	-9.4
PA0797	transcriptional regulator	-1.9	NC
PA0836 (*ackA*)	acetate kinase	1.9	NC
PA0962 (*dps*)	DNA-binding stress protein	2.3	NC
PA1300	RNA polymerase ECF-subfamily sigma-70 factor	NC	-28
PA1414	hypothetical protein	3.8	1.6
PA1429	cation-transporting P-type ATPase	3.9	-3
PA1546 (*hemN*)	coproporphyrinogen III oxidase	1.9	-4.8
PA1557 (*ccoN2*)	cbb3-type cytochrome c oxidase subunit I	3.5	-5.5
PA1561 (*aer*)	aerotaxis receptor	2.4	-1.5
PA1673	hypothetical protein	3.4	-5.5
PA1745	hypothetical protein	2.3	4.2
PA1746	hypothetical protein	3.5	-3.8
PA1920 (*nrdD*)	anaerobic ribonucleoside triphosphate reductase	2.4	-9.9
PA2126	conserved hypothetical protein	2.4	-1.9
PA2127 (*cgrA*)	hypothetical protein	1.9	-3.4
PA2575	hypothetical protein	2	NC
PA2753	hypothetical protein	4.4	-3.3
PA2754	conserved hypothetical protein	4.8	NC
PA3309 (*hepA*)	hypothetical protein	3.1	-1.7
PA3337 (*rfaD*)	ADP-L-glycero-D-manno-heptose-6-epimerase	2	-5.4
PA3391 (*nosR*)^**D**^	regulatory protein	NC	-4.4
PA3878 (*narX*)^**D**^	two-component sensor	NC	2.3
PA3879 (*narL*)	two-component response regulator NarL	1.7	1.6
PA3930 (*cioA*)	cyanide insensitive terminal oxidase	2	2.5
PA4205 (*mexG*)	hypothetical protein	1.9	NC
PA4328	hypothetical protein—response to stress	3.1	NC
PA4348	conserved hypothetical protein	NC	-5.5
PA4352	hypothetical protein—response to stress	4.8	-3.3
PA4577	hypothetical protein	4.2	NC
PA4587 (*ccpR*)	cytochrome c551 peroxidase	3.5	-17
PA4675 (*chtA*)	TonB-dependent receptor	-1.8	-5.1
PA5027	hypothetical protein	2.6	-2.8
PA5170 (*arcD*)	arginine/ornithine antiporter	2.7	NC
PA5171 (*arcA*)	arginine deiminase	6.8	NC
PA5172 (*arcB*)	ornithine carbamoyltransferase, catabolic	5.6	NC
PA5173 (*arcC*)	carbamate kinase	4.6	NC
PA5426 (*purE*)	phosphoribosylaminoimidazole carboxylase catalytic subunit	-2.1	-1.9
PA5427 (*adhA*)	alcohol dehydrogenase	5.8	-1.8
PA5475	hypothetical protein	3.8	-2.5
**Select genes encoding regulatory proteins implicated in virulence [35]**			
PA0652 (*vfr*)	cAMP-regulatory protein, virulence factor regulator	-1.7	-1.6
PA5550 (*glmR*)^**D**^	transcriptional regulator of polysaccharide biosynthesis	-2	-2.3
PA2426 (*pvdS*)	extracytoplasmic-function σ-70 factor	-2	-52
PA3879 (*narL*)	transcriptional regulator	1.7	1.6
PA5483 (*algB*)	two-component response regulator	3.6	NC
PA0610 (*prtN*)	transcriptional regulator	2.6	NC
PA3630 (*gfnR*)	glutathione-dependent formaldehyde neutralization regulator (sarcosine metabolism)	2.3	NC
PA5274 (*rnk*)	nucleoside diphosphate kinase regulator	-1.6	NC
PA0795 *prpR*	propionate catabolism operon regulator	1.8	NC
PA4080 *rcsB*	two-component response regulator, CupD activation	1.7	-1.6
**Regulators of swarming (of 20 total in S5 Table)**			
PA4853 (*fis*)^**1**^^**D**^	DNA-binding protein	-1.8	-2.1
PA0905 (*rsmA*)^**1**^^**D**^	RNA binding protein translational regulator	1.6	NC
PA4725 (*cbrA*)^**1**^^**D**^	two-component sensor	-1.5	NC
PA5261 (*algR*)^**1**^^**D**^	alginate biosynthesis regulatory protein	2.1	2.3
PA0762 (*algU*)	RNA polymerase σ factor	2.1	NC
PA2895 (*sbrR*)^**D**^	anti-σ factor	2.6	NC
PA2896 (*sbrI*)	RNA polymerase σ factor (inhibits swarming)	2.5	NC
PA3391 (*nosR*)^**D**^	regulatory protein	NC	-4.4
PA1713 (*exsA*)^**D**^	transcriptional regulator	-1.7	NC
PA4546 (*pilS)*^**D**^	two-component sensor (twitching, biofilm, swarming)	-1.7	NC
PA0479^**1**^^**D**^	LysR family transcriptional regulator	2.1	1.9
PA2072^**1**^^**D**^	sensory box protein	2	4.6
PA2571^**1**^^**D**^	signal transduction histidine kinase	1.7	4.2
PA1976^**1**^^**D**^	two-component sensor	2.5	NC
PA1196^**1**^^**D**^	transcriptional regulator	2.1	NC
PA1458^**1**^^**D**^	two-component sensor	1.5	NC

Gene expression is reported as fold-change (FC) relative to swarm front cells. Briefly, BM2 glucose swarm plates (± 0.75 μg/ml 1018) were inoculated with 1 μl of planktonic cells suspended at an OD_600_ of 0.4–0.6 in BM2 and then incubated for 20 h at 37°C. Cells were harvested from the leading swarming edge, the centres of swarming colonies, and 1018-treated colonies. NC = no significant change in gene expression.

^**1**^: Transcriptional regulators controlling swarming motility identified by Yeung *et al*. [36]

^**D**^: Swarming-deficient phenotype when mutated [11, 23, 36]

Compared to actively swarming cells from the leading edge of a swarming colony, the expression of Anr-regulated genes was different in peptide-treated and colony centre cells. A total of 141 of 253 known genes from the Anr regulon were dysregulated in either peptide-treated cells or cells from the centre of swarming colonies (S4 Table; see Table 1 for representative genes). Of these, 30 were dysregulated only by peptide treatment, 66 were dysregulated only at the swarm centre (44 of which were downregulated), 23 showed an opposite pattern of regulation between both conditions, and 22 showed similar regulation. Overall, treatment with peptide significantly triggered expression of the Anr regulon, as a total of 114 of these genes were more highly expressed in peptide-treated cells relative to untreated swarming cells. Gene expression of several other regulatory proteins that are involved in the stringent stress response, virulence and adaptive lifestyles (such as biofilm formation or swarming motility, both of which are inhibited by 1018 treatment) was influenced by peptide treatment but not by lack of motility at the centre of swarming colonies (Table 1, S5 Table). These virulence-associated or adaptation genes included *algB*, *algR*, *cbrA*, *prtN*, *gfnR*, *rnk* and *prpR*. Some of these genes encode regulators that are essential for swarming motility, such as CbrA [36]. Other swarming regulators for which transcription was altered by peptide treatment included the RNA-binding translational regulatory protein RsmA, as well as sigma and anti-sigma factors AlgU/MucA and SbrI [11, 36, 37].

### Serine Hydroxamate (SHX) enhanced the tolerance of certain mutants to 1018

**Fig 5:**
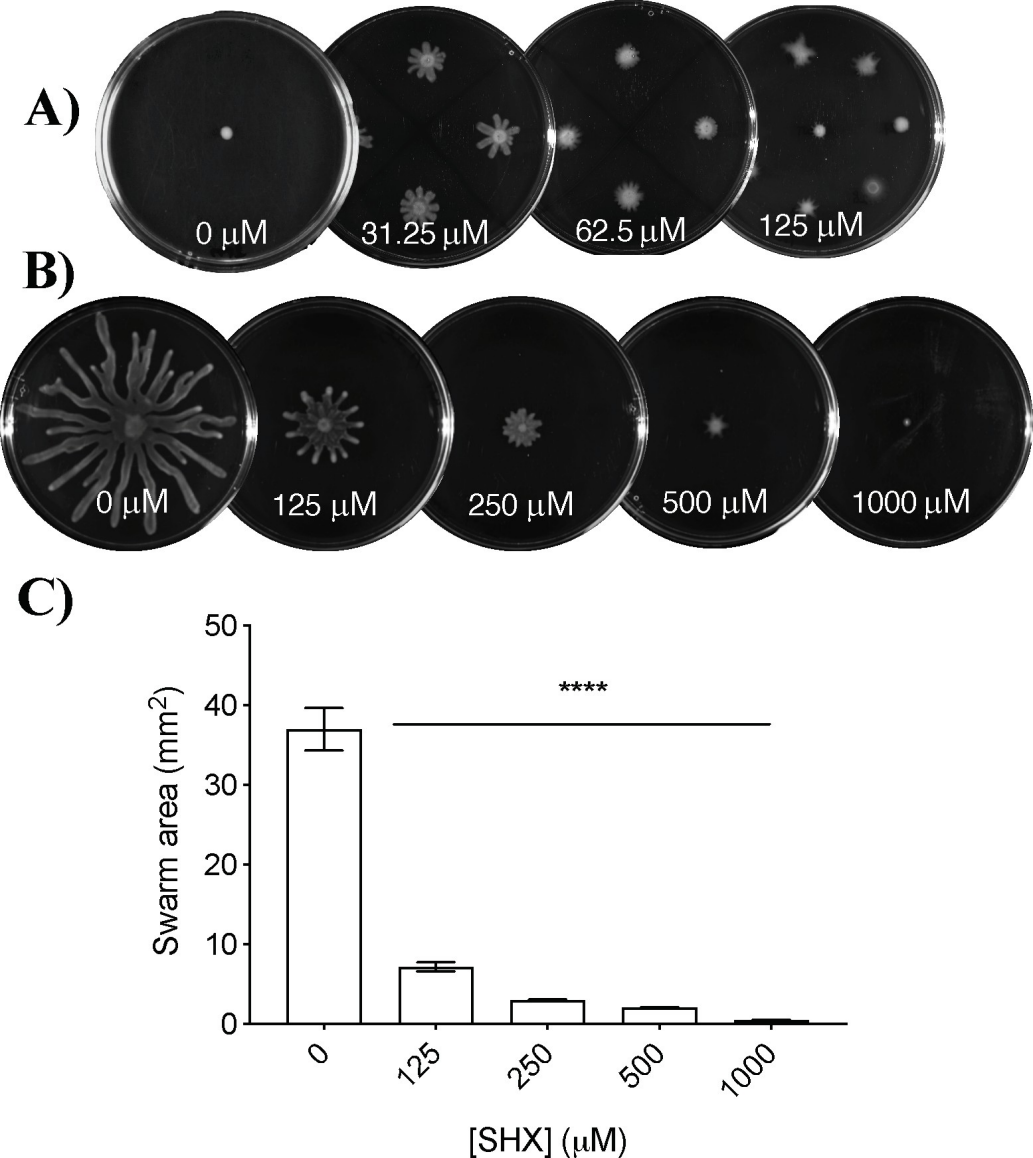
SHX partially restored swarming of peptide-inhibited cells in an inverse dose-dependent fashion and inhibited swarming of PA14 WT at higher doses. Briefly, BM2 swarm plates (±0.75 μg/ml 1018) were inoculated with 1 μl of planktonic cells suspended at an OD_600_ of 0.4–0.6 in BM2 and then incubated for 20 h at 37°C. The stringent stress response ((p)ppGpp production) was induced by supplementing swarm medium with SHX. (A) Peptide 1018 was antagonized by treatment with SHX at a low concentration (31.25 μM). (B) In the absence of peptide, SHX partially inhibited swarming at 125 μM. (C) Swarm colony area of SHX-treated cells (in the absence of peptide 1018). Mean and standard error of the mean are shown. One-way ANOVA followed by Dunn’s correction was used to determine statistical significance. **** *P* < 0.0001. Each experiment had at least three biological replicates.

It was previously shown that peptide 1018 inhibited biofilm formation by modulating the stringent stress response through direct interaction with (p)ppGpp promoting its degradation [21]. Thus, we examined the effect of peptide treatment on swarming in the presence of SHX, a structural analogue of L-serine that induces the stringent stress response by inhibiting seryl-tRNA synthetase from incorporating amino acids into proteins [38], leading to induction of (p)ppGpp synthesis (via RelA) in excess of 1018. Prior studies showed that SHX induced (p)ppGpp accumulation over a concentration range of ~50–500 μM [21, 38]. Here, we treated swarming cells with SHX in addition to 1018 (0.75 μg/ml) and observed that 31.25 μM SHX partially restored the swarming ability of WT cells in the presence of peptide (Fig 5A) without inhibiting swarming of cells in the absence of peptide. Although we observed that 62.5–125 μM SHX did not fully restore swarming in peptide treated cells, we also observed that SHX inhibited swarming motility of untreated cells in a dose-dependent manner (Fig 5B and 5C). These results indicate that high concentrations of SHX inhibited swarming motility independent of peptide treatment, which supports the importance of amino acid metabolism in swarming.

**Fig 6:**
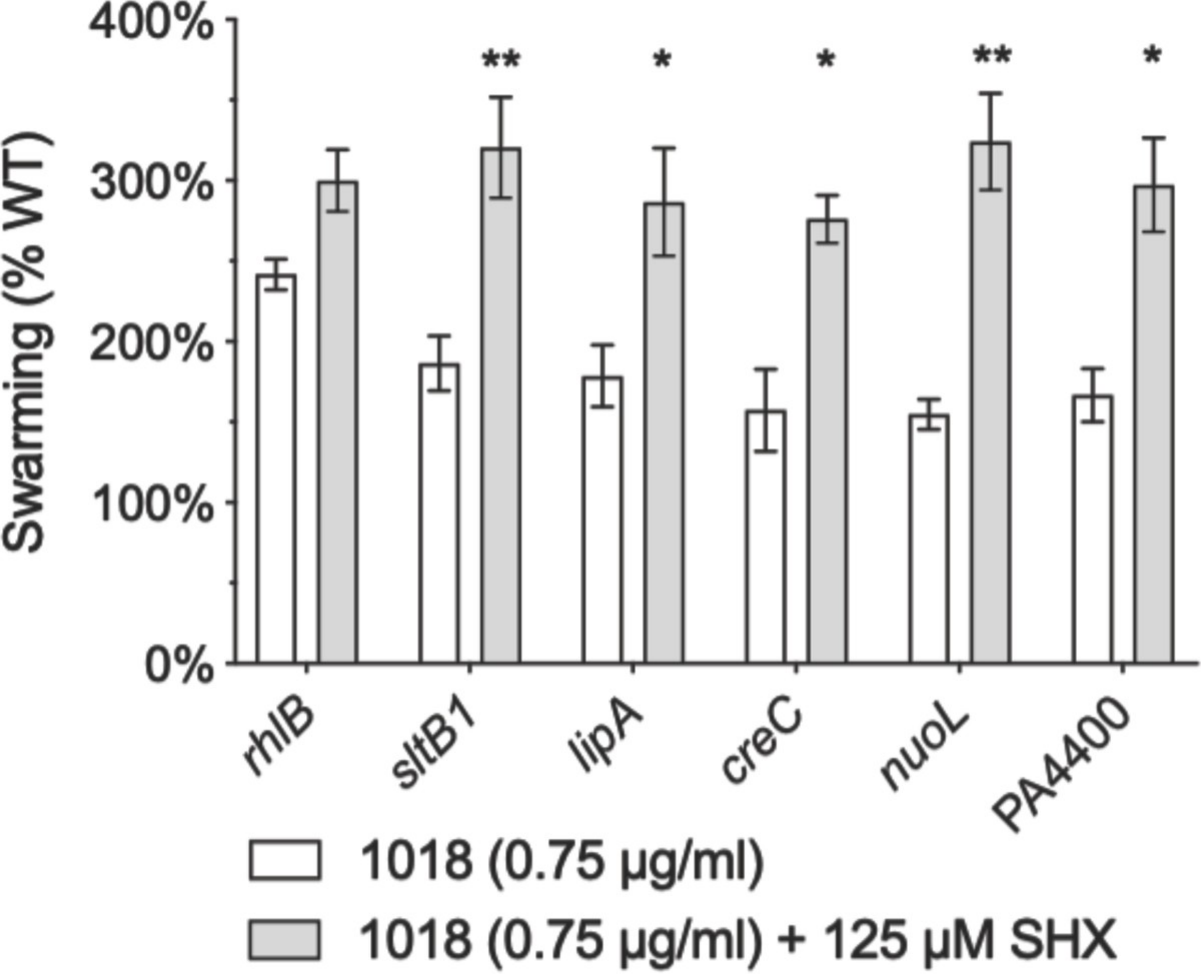
SHX enhanced the swarming phenotype of peptide 1018-tolerant transposon insertion mutants. BM2 swarm plates (+ 0.75 μg/ml 1018) were inoculated with 1 μl of planktonic cells suspended at an OD_600_ of 0.4–0.6 in BM2 and then incubated for 20 h at 37°C (A) Peptide-tolerant mutants showed enhanced swarming in the presence of SHX (125 μM). Mean and standard error of the mean are shown. Two-tailed student *t*-tests were used to determine statistical significance between treatments with or without SHX. * *P* < 0.05, ** *P* < 0.01. (B) Representative image of peptide-tolerant mutants swarming in the presence of SHX and peptide. Each experiment had at least three biological replicates.

Interestingly, the peptide tolerance of some transposon mutants (including *sltB1*::MAR2xT7, *creC*::MAR2xT7, *nuoI*::MAR2xT7 and PA4400::MAR2xT7) was even more pronounced in the presence of SHX (125 μM) and peptide (0.75 μg/ml) than it was in the presence of peptide alone, when compared to WT grown in respective conditions (Fig 6). The genes for these mutants encode a soluble lytic transglycosylase (SltB1) involved in cell wall biosynthesis [39]; the two-component sensor CreC, that together with CreB regulates β-lactam resistance, biofilm formation, fitness and anaerobic respiration [33, 40]; the NADH dehydrogenase NuoI; and a probable pyrophosphohydrolase PA4400 [30]. These data were consistent with the conclusion that these genes either had a role in the stringent stress response or worked in synergy with the stringent response.

## Discussion

Peptide 1018 is a known inhibitor of biofilm formation and can induce biofilm dispersion in a diverse range of species, including *P*. *aeruginosa*, at concentrations well below its MIC [21]. Here it was shown that 1018 completely inhibited swarming motility at a concentration of 0.75 μg/ml. This data aligned with observations from a prior study [11] that extended the screening to other *P*. *aeruginosa* motilities including swimming and twitching, and showed that neither were impacted by 1018 at concentrations up to 20 μg/ml. Building on our knowledge of 1018 as a swarming-specific inhibitor of motility, we identified a subset of genes that conferred partial peptide tolerance on *P*. *aeruginosa* under swarming conditions by screening a comprehensive transposon insertion mutant library. Similar effects have been observed when identifying genes that contribute to tobramycin resistance, where it was shown that 41 mutants contributed partially to adaptive resistance to this antibiotic [3]. We propose here that susceptibility to peptide 1018 is multigenic, whereby multiple genes collectively contribute to susceptibility. A number of the genes identified in the peptide tolerance screen have been implicated in SpoT-mediated activation of the stringent response. For example, LipA is a lipase involved in both fatty acid metabolism and the regulation of the iron-starvation sigma factor, PvdS [32]; the latter was two-fold downregulated in peptide-treated cells but strongly downregulated in swarming centre cells (S2 Table). Indeed, *lipA* expression is essential for full pyoverdine production under iron-limiting conditions and is lower in stringent response mutants [32]. Likewise, a sensor of carbon catabolites (CreC) activates PhoB, which regulates, through SpoT, adaptation to phosphate starvation in response to low levels of inorganic phosphate [33]. Inorganic phosphate is required for swarming motility in *P*. *aeruginosa*, and reduced levels are associated with motile-sessile switching and decreased expression of the T3SS [41]. KatB is a catalase induced by exogenous hydrogen peroxide and an important enzyme for stationary phase survival and antibiotic tolerance mediated by the stringent response [34]. Thus, peptide treatment might be inhibiting adaptations necessary for swarming motility since we showed peptide susceptibility could be reversed by inducing the synthesis of (p)ppGpp.

The data also provided evidence for a role of *anr* and *rhlB* in adaptation and tolerance to 1018 (S1 Fig). These genes were of particular interest because they modulate physiological processes associated with adaptive lifestyles such as biofilm formation, swarming motility, anaerobic growth, quorum sensing and the stringent stress response [11]. More specifically, *rhlB* is required for rhamnolipid biosynthesis, which is reduced in a non-swarming RelA/SpoT mutant [14]. Corroborating evidence was provided here, since *rhlA* and *rhlB* were 3-fold less upregulated in peptide treated swarming cells than untreated swarming cells when compared to the control (S2 Table).

The global regulator Anr influences metabolic processes that transform carbohydrates into energy across a range of oxygen levels [42, 43]. Our data revealed that treatment with peptide 1018 significantly triggered expression of the Anr regulon, since a total of 114 of these genes were more highly expressed in peptide-treated cells relative to untreated swarming centre cells. Intriguingly a connection has been made between the stringent stress response and *anr*, whereby the universal stress proteins are upregulated in *anr* mutants in a stringent stress response-dependent manner [44]. Furthermore, an *anr* mutant demonstrated a modest but complementable decrease in susceptibility to peptide 1018 under swarming conditions (Fig 3). This indicates that peptide 1018 works in part by modulating the Anr regulon to interfere with the stringent stress response. Consistent with this, some Anr-regulated genes that are required for swarming were dysregulated by peptide treatment, namely PA1196, *clpS*, *cspD*, *amrZ*, *nosR*, and PA4958 (S4 and S5 Tables), and *anr* deletion led to reduced swarming under anaerobic conditions [31].

There were significant differences between cells at the centre of swarming colonies and peptide treated cells that appeared in the same position of swarming plates. Indeed, the metabolically quiescent characteristics of centre cells did not appear to occur in the presence of peptide. Thus, transcripts for nitrogen metabolism genes including *nor* (nitric oxide), *nir* (nitrite reductases), and *nosR* (regulatory protein) were significantly downregulated (by 4.4- to 18-fold) in cells from the centre of swarming colonies, but not in peptide-treated cells (S2 Table). A similar trend was observed for transcripts encoding cytochrome oxidases (Ccp and Cco) that serve as terminal electron acceptors and are important for energy production [45]. Corroborating evidence was observed in genes implicated in amino acid (*argS*, *arcD*) and carbon (*rfaD*, *glcB*, *gltP*, *ackA*) metabolism as well as detoxification of reactive oxygen species (*dnr*, *katA*). No differences in expression for genes previously implicated in peptide resistance (*phoPQ*, *pmrAB*, *cprRS*, *parRS*, *arn* operon) or those important for adaptive resistance [3] were detected between peptide-treated cells and cells from the swarm front, suggesting that 1018 did not trigger canonical resistance mechanisms to peptides. However, the multidrug efflux pump *mexGHI-opmD* was upregulated by 1.8- to 2-fold in peptide-treated cells (S4 Table).

Examination of genes that were uniquely dysregulated in the presence of peptide revealed 50 other genes that are known to be required for swarming motility [37], including those for 19 regulators (Fig 3, Table 1, S4 Table). These included the regulatory genes *exsA*, *cbrA*, *pilS*, *glmR*, *fis* and the negative regulator *sbrI* (S5 Table). ExsA is a master regulator of the T3SS, and through interactions with the DNA-binding protein Fis has a variety of roles in optimizing bacterial adaptation and virulence [46]. T3SS is an important virulence factor in acute infections, and its expression is positively associated with swarming motility [10, 47]. The global regulator CbrA is involved in regulating swarming motility, biofilm formation, cytotoxicity against human bronchial epithelial cells, antibiotic resistance, and the adaptability of *P*. *aeruginosa* to nutritionally depleted environments [48]. The observed alterations in the expression of these genes and downstream effectors could collectively contribute to the inhibition of swarming motility by peptide 1018.

Evidence of the (at least partial) involvement of the stringent stress response was corroborated by observations that (p)ppGpp induction via SHX restored swarming in the presence of peptide (Fig 5) as well as enhanced swarming in the presence of peptide in some 1018-tolerant transposon insertion mutants (Fig 6). The alarmone (p)ppGpp is required for optimal swarming, and Δ*relA*Δ*spoT* double mutants, which lack the ability to synthesize (p)ppGpp, are deficient in swarming [11]. During biofilm formation, 1018 has been proposed to bind (p)ppGpp and mark it for degradation [21]. High levels of SHX inhibited swarming, likely because SHX inhibits the use of amino acids [21] that were supplied as the nitrogen source during swarming assays.

Preventing bacteria from adapting to their environment can make them vulnerable to antibiotic treatment and clearance by the immune system [48, 49]. As a complex and tightly regulated lifestyle adaptation, swarming motility confers fitness *in situ* by promoting mucosal surface colonization, dissemination to distal tissues [11], and antibiotic resistance [37], which can be blocked by 1018 treatment. Furthermore, *in vivo* models revealed that 1018 has protective immunomodulatory effects, including attenuated inflammation, immune cell recruitment, and enhanced wound healing and reduced bacterial spread [18, 20]. Therefore, peptide 1018 sensitizes bacteria to innate immune processes, bolsters these processes, and limits potentially harmful inflammatory responses. The data presented herein suggest 1018 as a promising anti-swarming compound that could be used in combination with anti-infective therapies to improve treatment of *P*. *aeruginosa* infections by inhibiting bacterial spread or adaptation.

## Supporting information

S1 Table List of strains and plasmids used in this study. (DOCX)

S2 Table Full list of differentially expressed genes, as determined by RNA-Seq, in peptide-treated and swarming colony centre cells under swarming conditions (FC ≥ ±1.5 and *P* ≤ 0.05). Gene expression is reported as fold-change (FC) relative to swarm front cells. Briefly, BM2 glucose swarm plates (±1018) were inoculated with 1 μl of planktonic cells suspended at an OD_600_ of 0.4–0.6 in BM2 and then incubated for 20 h at 37°C. Cells were harvested from tips of swarm tendrils, centres of swarming colonies, and 1018-treated colonies. 0 = no significant change in gene expression.
(XLSX)

S3 Table Full list of functionally enriched Gene Ontology (GO) terms, in peptide-treated and swarming colony centre cells under swarming conditions (FWER ≤ 0.1). Number of genes that were dysregulated for a particular GO term in each condition, as well as total number of genes annotated to that GO term, are shown.
(XLSX)

S4 Table Differentially expressed *anr* regulon genes in peptide-treated and swarming colony centre cells under swarming conditions, as determined by RNA-Seq (FC ≥ ±1.5 and padj ≤ 0.05). Gene expression is reported as fold-change (FC) relative to swarm front cells. Briefly, BM2 glucose swarm plates (±1018) were inoculated with 1 μl of planktonic cells suspended at an OD_600_ of 0.4–0.6 in BM2 and then incubated for 20 h at 37°C. Cells were harvested from tips of swarm tendrils, centres of swarming colonies, and 1018-treated colonies. 0 = no significant change in gene expression. References: [31, 35].
(XLSX)

S5 Table Differentially expressed essential swarming genes in peptide-treated and swarming colony centre cells under swarming conditions, as determined by RNA-Seq (FC ≥ ±1.5 and padj ≤ 0.05). Gene expression is reported as fold-change (FC) relative to swarm front cells. Briefly, BM2 glucose swarm plates (±1018) were inoculated with 1 μl of planktonic cells suspended at an OD_600_ of 0.4–0.6 in BM2 and then incubated for 20 h at 37°C. Cells were harvested from tips of swarm tendrils, centres of swarming colonies, and 1018-treated colonies. 0 = no significant change in gene expression.
(XLSX)

S1 Fig The peptide 1018 tolerant phenotypes of two transposon mutants, *anr-*Tn and *rhlB-*Tn were eliminated by complementation. (A, B) In the presence of peptide 1018 (0.75 μg/ml), *rhlB*-Tn and *anr*-Tn mutants swarmed more than the PA14 wild-type (WT). Non-swarming was restored by complementation of the respective gentic loci in trans since no differences were detected between complemented mutants (shown as mutant^+^) and WT transformed with empty vector (ev) (C, D) Representative images showing swarming phenotypes of mutants and complemented mutants in the presence or absence of peptide 1018, as indicated. Mean and standard error of the mean are shown. One-way ANOVA followed by Dunn’s correction was used to determine statistical significance. *** *P* < 0.001. Each experiment had at least three biological replicates.
(DOCX)
