# Supplementary material for: Peptide 1018 inhibits swarming and influences Anr-regulated gene expression downstream of the stringent stress response in Pseudomonas aeruginosa
Source: PLoS One. 2021 Apr 30;16(4):e0250977. doi: 10.1371/journal.pone.0250977 (PMC8087004; doi:10.1371/journal.pone.0250977)
Supplement: S1 Table — (DOCX) [file pone.0250977.s001.docx]

**S1 Table.** **List of strains and plasmids used in this study.** Transposon mutants were picked from the Harvard non-redundant (NR) transposon mutant library [22].

| Strain or Plasmid | Description |  |
| --- | --- | --- |
| PA14 WT | Laboratory wild-type *P. aeruginosa* strain PA14 |  |
| PA14 *rhlB::*MAR2xT7 | *rhlB* transposon mutant; Gen^R^ |  |
| PA14 *anr::*MAR2xT7 | *anr* transposon mutant; Gen^R^ |  |
| PA14_48920::MAR2xT7 | PA14_48920 transposon mutant; Gen^R^ |  |
| PA14 *nuoI*::MAR2xT7 | *nuoI* transposon mutant; Gen^R^ |  |
| PA14 *katB*::MAR2xT7 | *katB* transposon mutant; Gen^R^ |  |
| PA14 *rocA2*::MAR2xT7 | *rocA2* transposon mutant; Gen^R^ |  |
| PA14 *orfK*::MAR2xT7 | *orfK* transposon mutant; Gen^R^ |  |
| PA14 *ligD*::MAR2xT7 | *ligD* transposon mutant; Gen^R^ |  |
| PA14 *lipA*::MAR2xT7 | *lipA* transposon mutant; Gen^R^ |  |
| PA14 *creC*::MAR2xT7 | *creC* transposon mutant; Gen^R^ |  |
| PA14 *sppR*::MAR2xT7 | *sppR* transposon mutant; Gen^R^ |  |
| PA14 *ubiC*::MAR2xT7 | *ubiC* transposon mutant; Gen^R^ |  |
| PA14 *sltB1*::MAR2xT7 | *sltB1*transposon mutant; Gen^R^ |  |
| PA14_28800::MAR2xT7 | PA14_28800 transposon mutant; Gen^R^ |  |
| PA14_13530::MAR2xT7 | PA14_13530 transposon mutant; Gen^R^ |  |
