# Supplementary material for: Peptide 1018 inhibits swarming and influences Anr-regulated gene expression downstream of the stringent stress response in Pseudomonas aeruginosa
Source: PLoS One. 2021 Apr 30;16(4):e0250977. doi: 10.1371/journal.pone.0250977 (PMC8087004; doi:10.1371/journal.pone.0250977)
Supplement: S1 Fig — (A, B) In the presence of peptide 1018 (0.75 μg/ml), rhlB-Tn and anr-Tn mutants swarmed more than the PA14 wild-type (WT). Non-swarming was restored by complementation of the respective gentic loci in trans since no differences were detected between complemented mutants (shown as mutant+) and WT transformed with empty vector (ev) (C, D) Representative images showing swarming phenotypes of mutants and complemented mutants in the presence or absence of peptide 1018, as indicated. Mean and standard error of the mean are shown. One-way ANOVA followed by Dunn’s correction was used to determine statistical significance. *** P < 0.001. Each experiment had at least three biological replicates. (DOCX) [file pone.0250977.s006.docx]

**
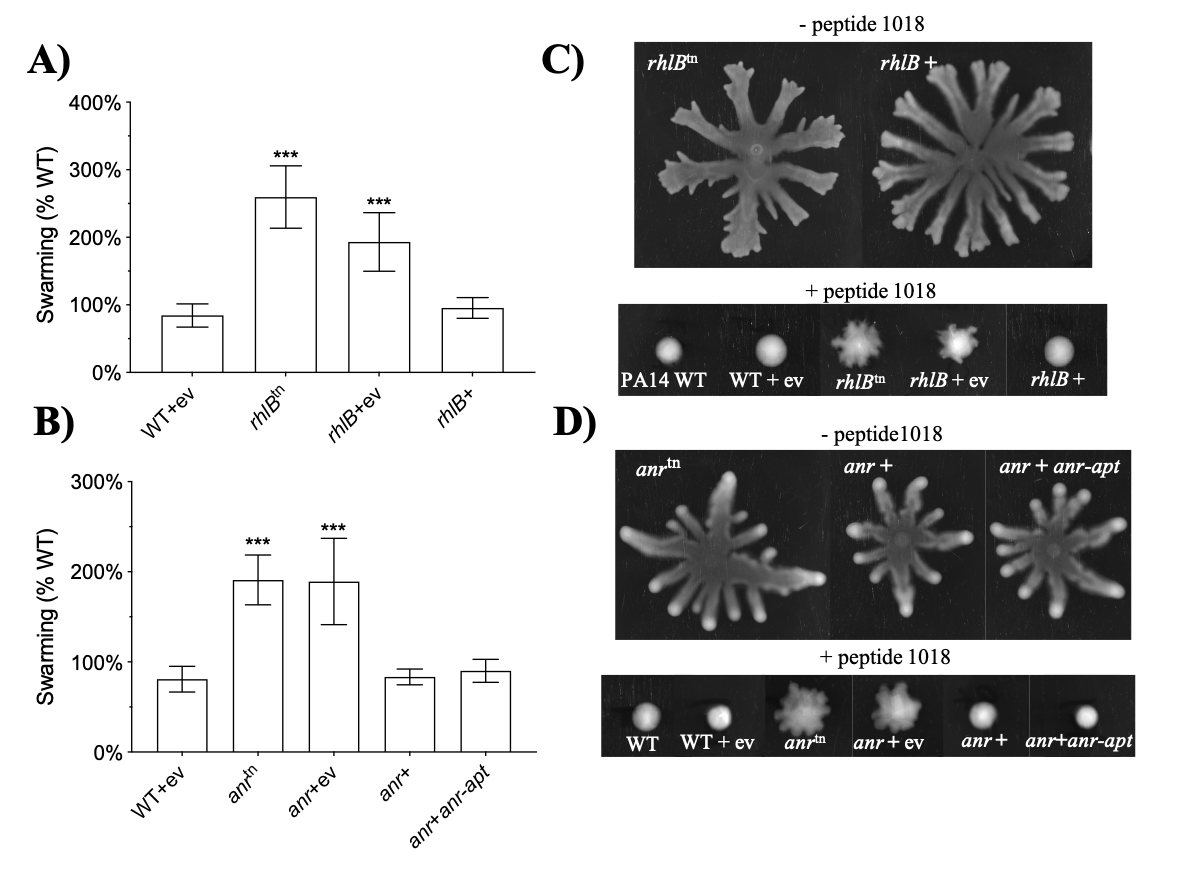
**

**S1 Fig**. **The peptide 1018 tolerant phenotypes of two transposon mutants, *anr-*Tn and *rhlB-*Tn were eliminated by complementation. (A, B)** In the presence of peptide 1018 (0.75 μg/ml), *rhlB*-Tn and *anr*-Tn mutants swarmed more than the PA14 wild-type (WT). Non-swarming was restored by complementation of the respective gentic loci in trans since no differences were detected between complemented mutants (shown as mutant^+^) and WT transformed with empty vector (ev) **(C, D)** Representative images showing swarming phenotypes of mutants and complemented mutants in the presence or absence of peptide 1018, as indicated. Mean and standard error of the mean are shown. One-way ANOVA followed by Dunn’s correction was used to determine statistical significance. *** *P* < 0.001. Each experiment had at least three biological replicates.
